# Successful artificial reefs depend on getting the context right due to complex socio-bio-economic interactions

**DOI:** 10.1038/s41598-021-95454-0

**Published:** 2021-08-17

**Authors:** Timothée Brochier, Patrice Brehmer, Adama Mbaye, Mamadou Diop, Naohiko Watanuki, Hiroaki Terashima, David Kaplan, Pierre Auger

**Affiliations:** 1 UMMISCO, Sorbonne Université, SU, Institut de Recherche pour le Développement, IRD, F-93143 Bondy, France; 2 UMMISCO, Université Cheikh Anta Diop, UCAD, Ecole Supérieure Polytechnique, ESP, IRD, BP 15915, Dakar, Senegal; 3 Ifremer, CNRS, IRD, Univ Brest, Plouzané, Lemar France; 4 Centre de Recherche Océanographique de Dakar-Thiaroye, CRODT, Institut Sénégal de Recherches Agricoles, ISRA, Dakar, Senegal; 5 Directorate of Community Marine Protected Areas, DAMCP, Ministry of the Environment and Sustainable Development, MEED, Dakar, Senegal; 6 OAFIC, 2F Kanda 4th Amelex Bldg., 2-13 Kanda Tsukasamachi, Chiyodaku, Tokyo 101-0048 Japan; 7 IC Net Limited, Land Axis Tower 27th Floor, 11-2 Shintoshin, Chuo-ku, Saitama, 330-6027 Japan; 8 MARBEC, Univ. Montpellier, CNRS, Ifremer, IRD, Sète, France; 9 IRD, MARBEC, av. Jean Monnet, CS30171, 34203 Sète cedex, France

**Keywords:** Ecological modelling, Environmental economics, Psychology and behaviour, Socioeconomic scenarios, Sustainability

## Abstract

Artificial reefs (ARs) are one of the most popular means of supporting marine ecosystem conservation and coastal fisheries, particularly in developing countries. However, ARs generate complex socio-bio-economic interactions that require careful evaluation. This is particularly the case for ARs outside no-take zones, where fish might be subject to enhanced exploitation due to easier catchability. Here, we conducted an interdisciplinary study on how ARs impact fish and fishing yields, combining mathematical and sociological approaches. Both approaches converge to confirm that fishery yields decline when ARs are exploited as if they were open access areas. This situation typically occurs in areas with weak governance and/or high levels of illegal fishing activity, both of which are common in many developing countries. To avoid these adverse effects and their associated ecological consequences, we recommend prioritizing the onset of a long-term surveillance system against illegal fishing activities, and adapting design and location of the ARs based on both and local and academic knowledge, before the deployment of ARs.

## Introduction

Coastal artisanal fisheries, particularly those in developing countries, are facing a global crisis of overexploitation^1^. Artificial reefs (ARs), or human–made reefs^2^, have been widely advocated by governmental and non-governmental conservation and management organizations for addressing these issues. Industries, particularly oil and gas, seeking to avoid the costs of removal or conventional disposal of used materials are often major advocates for deploying ARs. Yet, major questions remain regarding the success of such efforts in the context of weak governance and poorly sustained international investment in AR development projects. There is frequently confusion over whether or not ARs should be fishing sites and the precise goals of constructing such ARs are often unclear, making difficult to evaluate their successfulness^3^. Over the last 40 years, both failures and success AR implementation programs have been reported^4,5^. The main point of the present work is to underline the importance of the governance issue and address social and management factors on AR “success”.

To improve fishery yields, it has been recommended that ARs must be no-take areas (*e.g*.,^2^). Yet, most ARs were historically delineated as sites for fishing^4^, and were rarely implemented at large scales in/for no-take zones, even in countries with centuries of experience in constructing ARs, such as Japan. In Japan, fishery authorities and local fishers use ARs to promote sustainable catches and to establish nursery grounds of target species^6^. However, fishery authorities and local fishery cooperatives in Japan have extensive management authority over ARs. For example, fishing around ARs is usually limited to hook and line techniques, with net fishing rarely being permitted in areas where risk of entanglement in ARs is high. Furthermore, during spawning, fishing gear and fishing season are often restricted around ARs in Japan. These practices are recognized for their effectiveness in maintaining good fishing performance and marine conservation in Japan and elsewhere where they have been implemented^7^.

Attempts to transpose ARs to developing countries have, however, frequently ended in failure^8^, particularly when project funding comes to an end^9^. Thus, it is important to provide recommendations to improve the sustainability of AR deployments and realize their biodiversity conservation and fisheries management goals. This is particularly important in developing countries, which are often characterized by poor governance. For fisheries scientists and marine ecologists, the effectiveness of ARs is primarily quantified by surveying fish populations on ARs. In particular, the question of whether ARs facilitate the “production” of new fish or whether they only attract the surrounding fish remains under debate^10–12^. Few studies have documented how ARs are managed, and the impacts of such management^8,13^, despite the key importance of protecting no-take ARs from illegal fishing being repeatedly highlighted^2^. Mathematical models, implemented to set the optimal AR volume to maximize catches, suggest that, although attraction and production effects can modulate the response, the effect of ARs on fisheries mostly depends on governance options and efficiency^14^. Existing models show that fishing exclusively on ARs has consistently negative impacts on the equilibrium of catches. In comparison, ARs can have negative or positive impacts on catches when fishing on areas surrounding them, as a function of the magnitude of the AR attraction effect^14^. Whether or not ARs are managed as no-take areas influences these phenomena. For instance, on unmanaged ARs, overexploitation risk increases, as fish become more accessible to fishing fleets. In comparison, when fishing is banned on ARs, the fish biomass concentrated near the AR rises, leading to a “spill-over” effect that enhances catch at equilibrium in adjacent fishing areas^15^.

**Figure 1:**
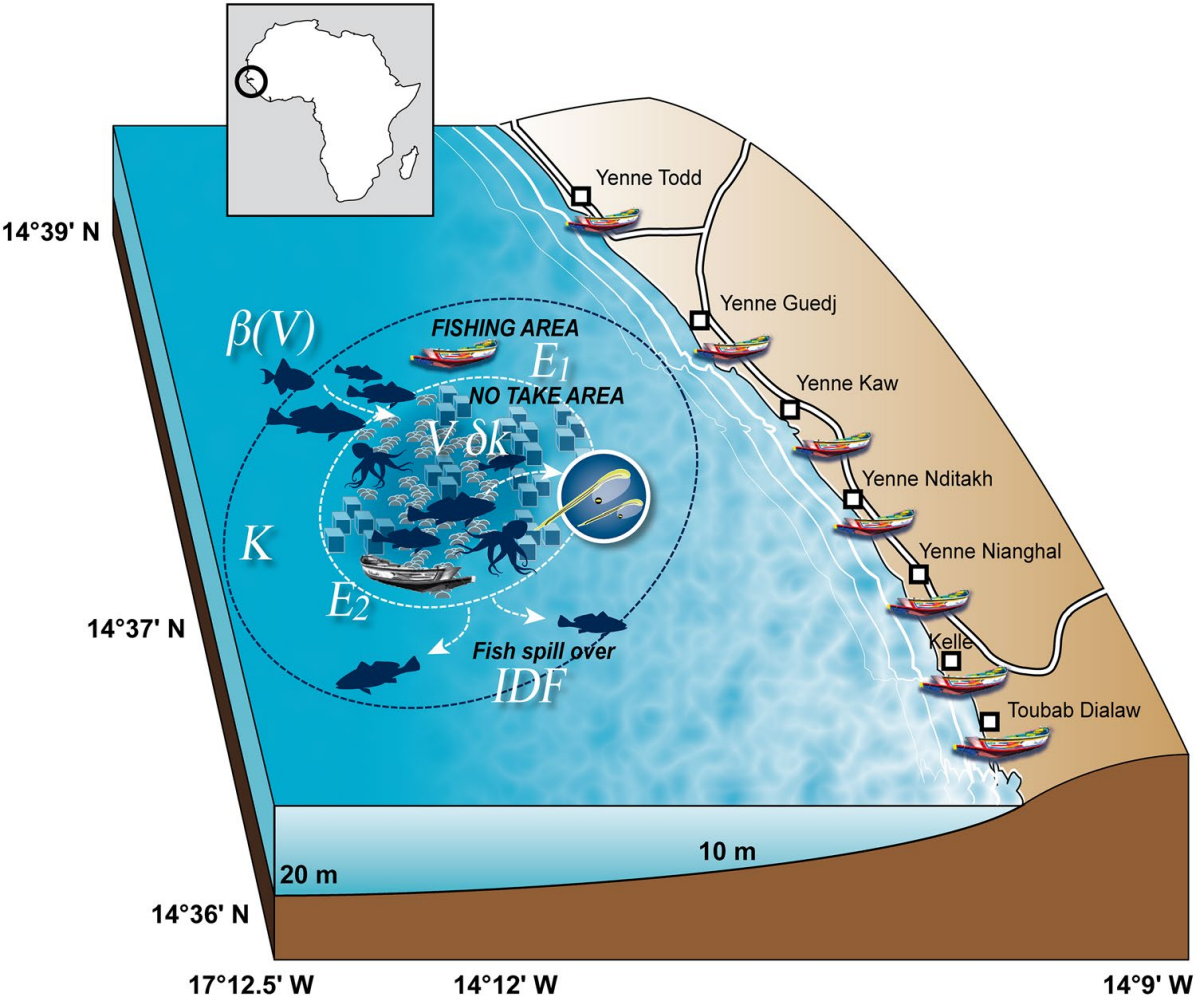
Map of the artificial reef off Yenne, Senegal (West Africa), showing the seven fishing villages surveyed that regularly operate around the artificial reef. The key processes that were investigated are summarized in this conceptual scheme. K (tons) is the carrying capacity of the study area which comprises the fishing area and the no take area (prior artificial reef deployment); ∂k is the supplementary carrying capacity (fish growth and reproduction) per artificial reef volume (V in m^3^) deployed in the no take area, *i.e*., effect of fish production; ß(V) is the effect of fish attraction; E_1_ and E_2_ area the fishing effort applied resp. in the no-take area and fishing area, *i.e*., E_2_ is the illegal fishing effort. The spill-over effect is a consequence of the ideal free distribution (IDF) in fish behavior between the fishing area and no take area. This figure was generated by Pierre Lopez (IRD) using Adobe Illustrator CS3 and Adobe Photoshop CS2.

Here we assumed that ARs and marine reserves behave as is generally accepted for fish attraction, production, and, ultimately, spillover^10,11,15^. Thus, the ecological “performance” of ARs was not explored. Instead, we focused on establishing whether the impact of ARs on the ecosystem and associated fisheries was strongly dependent on social and management factors. To explore this, we combined a bio-economic model with a social survey of fishers. Both approaches were designed to understand how the governance framework and illegal fishing impact AR benefits. We used a case study in Senegal (West Africa, Fig. 1), which is an area typical of coastal fisheries in developing countries, with a growing fisher population, fishery over-capacity, ecosystem perturbations due to warming waters, weak local governance, and little government investment to maintain international or non-governmental organization (NGO) AR initiatives.

The case study was a pilot project initiated by the Japanese International Cooperation Agency ‘JICA’ in 2004^16^. The purpose was to regenerate fishery resources and to introduce a resource management structure adapted to the local community. As part of the project, an artificial reef composed of 75 cubic concrete blocks 400 gabions were built by the local community members to foster AR ownership by the community and deployed in the coastal zone south of Dakar, in the neighborhood of 7 fishers villages (Fig. 1). The total volume of the AR reef was ~ 70 m^3^ (30 m^3^ of concrete cubes and 45 m^3^ of gabions) and the area covered by these structures was about 550 m^2^. Simultaneously, a local management committee, constitute by small-scale fishers, was set up with the support of JICA and has endeavored to manage the area around the ARs. Subsequently, underwater monitoring was carried out for two years and the effect of fish attraction around the AR was quantified^16^, confirming the testimony of local fishers. Since then, several research and management projects focused on this AR provided detailed biological and socio-economic data in the years following its immersion^14,17^, but no systematic or quantitative survey has been conducted so far. However, after the project ended, which funded the monitoring and surveillance, because the AR attracted both fish and fishers, local overfishing and fisher conflicts were reported.

In this context, we specifically sought answers on whether current AR projects in developing countries sufficiently address the social and governance framework. Based on results, we propose some general recommendation necessary for ARs projects in developing countries to be useful and effective for fishery management and conservation.

## Results

### Effect of illegal fishing on artificial reef

**Figure 2:**
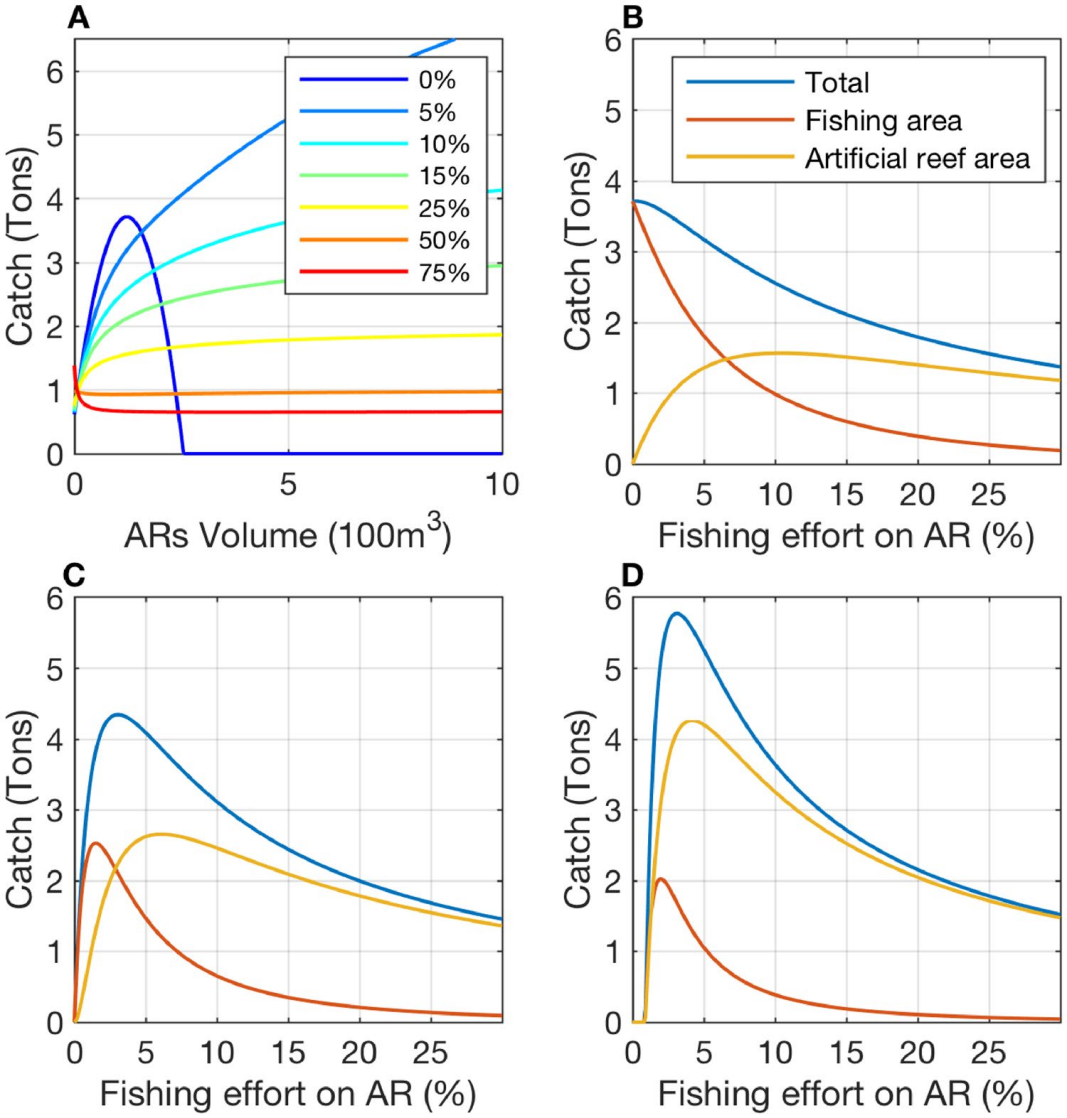
Mathematical model predictions of the catch resulting from interaction between the volume of artificial reef (AR) and fishing effort. (**A**) Colored curves correspond to different levels of (illegal) fishing effort across the ARs; the gradient from dark blue to orange represents 0, 5, 10, 15, 25, 50 and 75% of the total fishing effort. (**B**–**D**) Blue, red, and yellow curves correspond to total catch, catch in the fishing area, and catch on the AR, respectively, which is 120, 200 and 500 m^3^ respectively in subfigures B and C. Note, for zero (0%) illegal fishing, there is an optimal volume of AR (**A**); below this optimal volume, increasing illegal fishing decreases catch (**B**); in contrast, for higher AR volume, there is an optimal level of illegal fishing (**C**). The artificial reefs used in the model have production (δk = 5) and attraction (β_0_ = 1) effects (see complete analysis of the mathematical model in the supplementary text). Sensitivity tests with different artificial reef production and attraction levels are presented in Fig. S1–S3.

The model showed that, in the absence of illegal fishing, there was an optimum volume of ARs to maximize catch in ARs with both production and attraction effects (120 m^3^, Fig. 2A). In contrast, when illegal fishing was present but < 25%, total catch (legal and illegal) increased with increasing AR volume; however, when illegal fishing was > 50%, total catch declined with increasing AR volume (Fig. 2A). Consequently, large ARs with > 25% illegal fishing provided much lower total catches compared to smaller ARs with less illegal fishing (Fig. 2A).

If AR volume was below 120 m^3^ (optimal volume in the absence of illegal fishing), all levels of illegal fishing would cause total catch to decline, and catch became larger on the AR than in surrounding open access fishing areas if illegal fishing exceeded 6% (Fig. 2B). If AR volume was between the optimum and 256 m^3^, catches were highest with a small illegal fishing rate (~ 3%), and most of the catch was derived from the fishing area (Fig. 2C). For larger AR volumes, the fishery was not economically viable if access to the AR was prohibited (Fig. 2A). For an AR of 500 m^3^, a minimum illegal fishing rate of 1% is necessary, and the maximum catch is reached for 3% illegal fishing, with the majority of the catch coming from illegal fishing (Fig. 2D).

**Figure 3:**
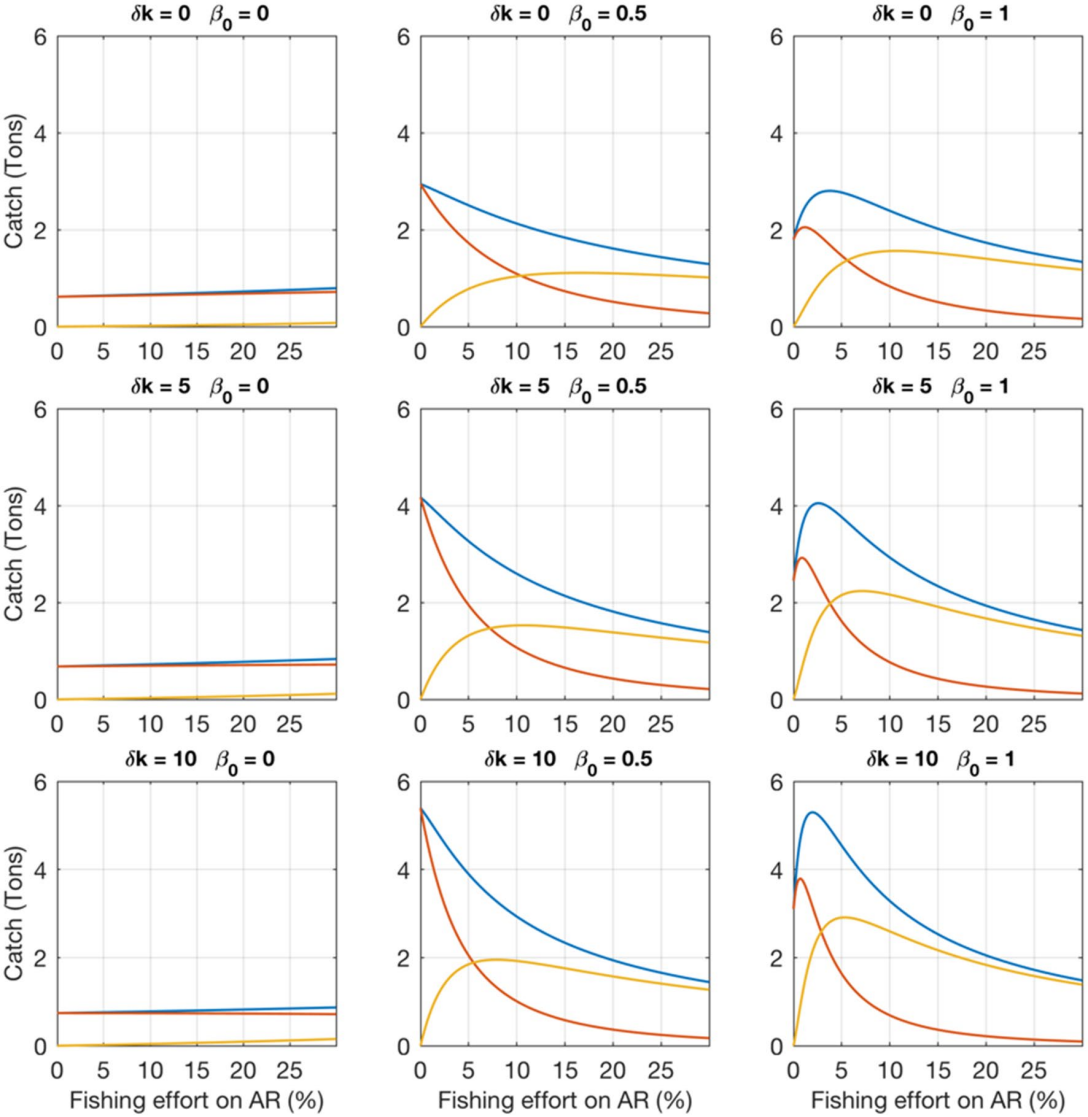
Effect of variable AR production and attraction on catches predicted by the mathematical model (Y-axis) for different levels of fishing effort on AR (X-axis). Images from left to right correspond to an increasing AR attraction parameter, and from top to bottom correspond to an increasing production parameter. Blue, red, and yellow curves correspond to total catch, catch in the fishing area, and catch on the AR, respectively, which was constant (200 m^3^).

Sensitivity tests showed that fish attraction was the main parameter driving the dynamics. In the absence of an attraction effect, AR production minimally affected catch, regardless of illegal fishing rate (Fig. 3, left column). In contrast, when AR attraction was low, total catch was enhanced, even though catches in the fishing area rapidly declined with increasing illegal fishing activity (Fig. 3, central column). For higher attraction effects, a maximum catch was derived for small (illegal) fishing effort on the AR (Fig. 3, right column). For any value of the attraction parameter, increasing the production parameter increased both legal and illegal catches, but had much weaker effects than attraction. Details on the interactions of the rate of illegal fishing with attraction and production effects are provided in the Supporting Information (Figs. S1–S3).

Model sensitivities to the value of other model parameters were explored by fixing the AR volume, production and attraction parameters and then varying remaining parameters individually. Increasing the population growth parameters r and K uniformly increased the catch (Fig. S4). Increasing the cost of fishing effort also increased the catch, while the opposite occurred for fish price, i.e. the lower the fish price, the higher the catch. For a combination of high cost of fishing effort and low fishing price, the fishery was not viable if less than 2% of fishing effort on the AR was allowed (Fig. S5). Reducing the fish catchability increased catch at equilibrium, and increasing the fish mobility parameter either increased or decreased the catch depending on whether or not the catchability is small or large (Fig. S6).

### Fisher perceptions

The main reason cited for declines in catches was overfishing (95%). Respondents stated that overfishing was mainly caused by both local and illegal industrial fishing boats (70%) operating (illegally) in coastal areas, and to a lesser extent to artisanal fisheries (30%). This answer could be naturally biased by the fact that respondents were artisanal fishers. Habitat destruction was cited by 21% of respondents, who mainly mentioned industrial bottom trawling as being responsible. None mentioned climate change as a reason for fish declines. However, 61% of fishers suggested other reasons for fishery declines, including the use of specific fishing gear (mainly monofilament purse seine nets) and fishing activities targeting juvenile fish. Overall, 72% were in favor of fisheries management rules being implemented, 63% were in favor of the concept of no-take areas, and 92% were in favor of limits to catch volume. However, only 39% would accept rules limiting the size of fish captured. At the local scale, 81% of respondents perceived an increase in biomass in the AR directly after its deployment, 14% stated that biomass was stable or did not know, and 4% stated that biomass had decreased. Overall, 46% of respondents thought fish were attracted to the AR (hydrodynamic effects and visual cues were the most cited reasons), 53% thought that fish find the AR by chance, and only one fisher had no opinion. To the question “to who belong the fish on the AR?”, all fishers (100%) answered “to all Senegalese fishers,” in preference to all other possible answers, which were local fishers or the state. Fishers did not provide information about the location of their fishing sites, except that their offshore limit was 3 to 10 km. Fishers did not clearly answer the question about the presence of migrant fishers, with some explaining that Yenne fishers were, themselves, migrants. Many targeted fish species were cited, including predatory and prey pelagic and demersal fish species, plus lobsters, sharks, cuttlefish, and octopuses. To the question of which community was fishing on the AR, only 4 (5%) fishers stated that no one was fishing there. All other interviewed fishers (95%) stated neighboring communities or that, in absence of surveillance, all fishers from Yenne were fishing on it. Respondents were not comfortable with the question about the existence of conflicts. Only 14% stated the existence of conflict over AR usage, mainly between line and net fishers and with neighboring communities. However, impressive stories of open conflicts around the AR involving numerous fishers were reported once the questionnaire was finished.

Most fishers (80%) considered that the AR was actually an open access fishing area, contrary to the official position of the national authority and local managers. However, the same fishers considered that the AR could only be useful to them if fishing was banned on it. Fishers who advocated the banning of fishing activities on the AR had an empirical understanding of fish population dynamics, which allowed them to anticipate the beneficial effects of this practice. Their opinion, expressed in their answers to the open questions, was based on empirical knowledge that fish concentration on the AR might favor either their reproduction and/or growth if protected from local high fishing pressure. The latter also caused a feeling of inequity among fishers, depending on their ease of access to the AR for fishing.

**Figure 4:**
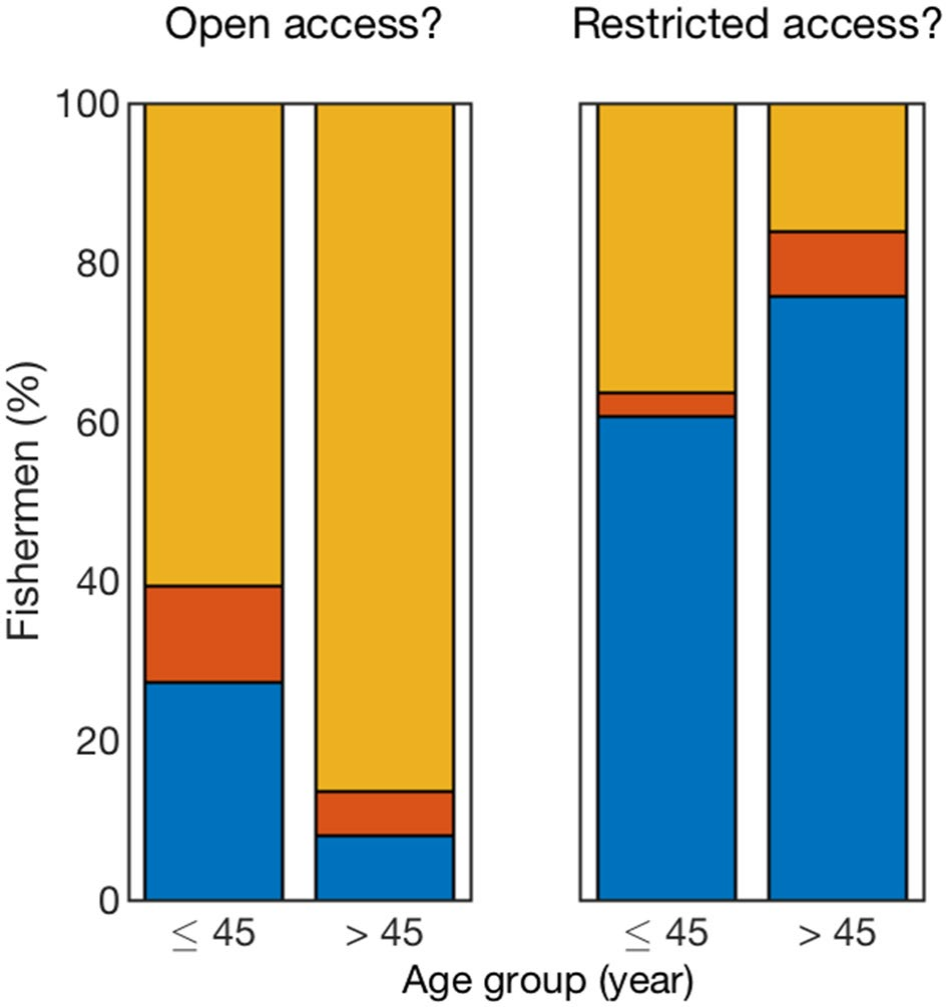
Distribution of fisher answers to two survey questions. (**A**) Is open access to the artificial reef by all fishers useful for fisheries management? (**B**) Is restricted access to the artificial reef a useful fisheries regulation measure? Blue, red, and orange are the percentage of fishers that answered “useful”, “useful if temporary,” and “useless”, respectively. There was a significant difference in how fishers perceived the usefulness of the artificial reef according to their level of experience. This phenomenon was also related to their understanding of fish population dynamics and fish movement and behavior (assuming that the level of experience is proportional to the age of fishers).

To further investigate the contrasting perceptions of fishers and how the AR was actually used, we clustered the surveyed fishers by age. The older group, named “experienced fishers” (older than 45), were more involved in co-management (Fig. S6). This included delegating government responsibility to Fishers’s organizations and fostering the bottom-up approach of fisheries management. Most of the more experienced fishers (86%) considered the open access regime of AR useless as a fisheries management tool (Fig. 4). Over half of younger fishers (61%) shared this perception, of which several (27%) were convinced of the usefulness of open access ARs. The justifications for their position involved the higher short-term economic needs of younger fishers, and a lack of awareness of the consequences of failing to consider the basic concepts of fish population dynamics. These perceptions might also be linked to their lower involvement in co-management (Fig. S6). Intermittent fishing on ARs was suggested by a large component of the fishers, mostly aged 45 years and younger (Fig. 4).

## Discussion

When introducing ARs as a fisheries management tool to Senegal, the Japanese management had the mindset of Japanese stakeholders, *i.e*., introducing fishing rights. However, after discussions with Senegalese stakeholders, it was decided that no-take areas would be delineated around ARs because the establishment of a strong fishing rights regime was not socially acceptable to the Senegalese fishing community. Japanese governance is based on the acceptance and respect of fishers towards individual, private AR concessions. In contrast, fishers in Senegal, and more widely in West Africa, are characterized by high mobility, particularly in the context of climate change and overexploitation^18,19^. Consequently, respect for local management regulations is lower, with open access being generally assumed. The basic concept of implementing a no-take area on the AR was not easily accepted by fishers. The immersion of AR concrete blocks was set as a top priority by managers at the expense of more complex socio-economic considerations, such as consciousness-raising activities and self-sustaining participative monitoring of the AR.

The clear contradiction between the ecological knowledge of fishers and their behavior was explained by the well-known effects of open access resources on individual behavior. This phenomenon was also observed in our mathematical model. The processes in the mathematical model are in accordance with those perceived by the fishers, so that the results are also those expected by fisher’s local ecological knowledge. It is interesting to notice that the theoretical results presented here are the mathematical solutions of the model at equilibrium between fishing effort and fish population growth, i.e. after an oscillation period. It is obvious that short-term effect of fishing on the AR is always to increase the catch, but many fishers did perceive the longer-term effect of decreasing catches. The potential negative effect of the AR on catch when there is high fish attraction combined with high fishing pressure on the AR might explain the reluctance of a part of the fishers community to AR deployment (Fig. 2). In particular, the model illustrates that the AR attraction effect strongly determines the impact of the management. In general, fish attraction is the most immediate effect perceived after AR deployment^11^, as was true for our study^16^. Though the AR volume was relatively small (70 m^3^), the empty space between the higher blocks also contributes approximately 280 to 570 m^3^ of good habitat/refuge for schooling fish; therefore, it is actually difficult to accurately describe the volume that affects fish. Thus, it is difficult to say whether this AR is below or above the forecasted optimal volume in absence of fishing (120m^3^ with model parameters). The existence of an optimal volume for AR was also suggested by field studies as a trade off between food supply and refuge^20^, in line with our results. For management purposes, it is interesting to determine whether the AR is above or below this optimal level because if the volume is too small, the model predicted that any level of fishing on the AR would, in the long term, decrease the catch in the considered area. On the other hand, if the volume is above the optimal level, a small fishing effort on the AR could be authorized and would increase the total catch in the area.

Field observation showed that the fish attraction effect was strong^16^ but precise estimation of this parameter cannot be inferred, as this would need, ideally, individual fish trajectories. Future field research on the attraction effect may permit estimating the AR attraction parameters. The model sensitivity test showed that the stronger the attraction parameter, the better the impact of the AR for the fisheries in case of no or small fishing effort on the AR (Fig. 3). But at the same time, the attraction is a strong incentive for fishers to fish on the AR, and the predicted benefit for fisheries in the fishing area rapidly vanishes when fishing effort on AR increases. This in turn provides further incentive for fishers to fish the AR, challenging the surveillance capacity. If fish attractiveness is strong and too many fishers fish on the AR, catch in the area will be concentrated on the AR, while the adjacent fishing area will be depleted, with catch levels lower than those prior to AR deployment.

Specifically, in the context of generalized overfishing in Senegal^21^, deciding not to fish on the AR represents significant individual loss, despite being recognized as beneficial, globally^22^. It has been argued that this situation would rarely occur in small-scale fisheries, due to existing arrangements between individuals^23^. However, in the context of the highly mobile Senegalese artisanal fishing fleet and its overcapacity, as soon as the AR in Yenne was no longer subject to surveillance, it rapidly attracted fishers from other villages. Also, pre-existing arrangements between fishers might be overruled when new ARs are created, changing the structure of existing fishing grounds.

At the time of the survey, the surveillance system set up by the co-management entities was not operational in our case study, because it was dependent on temporally limited external financing. These limitations are typical of short-term projects that focus on a single restricted area for a pre-determined duration, usually up to two years (*e.g*., NGOs, World Bank). Local fishers perceptions were globally in line with the model prediction that this AR fails to improve fisheries yield when surveillance is not in place to ensure AR regulations are observed, despite effective fish attraction and production existing in the AR.

The model predicted that enhanced production on ARs could not keep pace with unrestricted access, which might be particularly true in Senegal where fishing effort rapidly reorganizes itself according to local yields^24^. Enhanced production due to the AR largely increases the catch if the fishing pressure on the AR remains null or very low, but it has no effect on the catch for higher fishing pressures on the AR (Fig. 3). These results were stable even if fish population growth, fish catchability, mobility and economic parameters could modulate the predicted amplitude of the catch and AR optimal volume. These results are consistent with existing theoretical studies of the impact of fisher movement to high production areas in and around MPAs^25^. Taking into account several species and their interactions (predation, competition) would lead to a very complex ecosystem model specific to the area (e.g. ^26^), with necessarily more assumptions. This model would necessarily be more difficult to share with fishers and other stakeholders. Both to simplify model structure and facilitate communication of results to stakeholders, we assumed in our model that the balance of entries exits and is in equilibrium, so that the migratory species did not affect the long-term equilibrium between fishing effort and fish abundance.

The design of ARs could be adjusted to reduce the effect of illegal fishing by passively preventing both industrial and artisanal fishing activity. Complex structures are more effective for fish production and attraction^27^. We showed that, although production might have a limited effect on total catch, attraction can largely increase AR efficiency (total catch) if the rate of illegal fishing rate is very low or absent. Complex structures protect fish more effectively from small scale fishing gear^28^, including divers (*Pers. Comm*., Mamadou Sarr, Ouakam fishers committee). Thus, ARs should be appropriately designed to help mitigate potential issues^28^. Such designs might be more costly, and do not exclude the need for surveillance, but would enhance fisheries management, especially when surveillance cannot capture low levels of illegal fishing.

Finally, if socio-economic and governance conditions are not met, well-intentioned AR projects will likely disturb the existing equilibrium among fishers that have different levels of access to the AR. Poor governance of marine resources has previously been described in West Africa, particularly in Senegal^29^, as has the failure of AR projects in a number of other developing countries^9^, which further deteriorate fishers trust and management plans efficiency^30^. In order to avoid that, NGO and governmental agencies driving ARs projects must consider that AR management induces collective costs before providing potentially collective gains. Thus, co-management that involves governmental institutions and fisher communities is required. Future management and adaptation plans for fishers, particularly in developing countries, should, therefore, focus efforts on raising long-term awareness of actors in both government institutions and fishing communities. At the level of institutional or development partners, long-term management costs should be included in the set-up of AR projects. For example, the local fishers committee of Yenne recently reported the establishment of a collective ship chandler whose profits are used to finance AR surveillance during the daytime. Subsequently, fishers noted an improvement in catches around the AR, even though illegal fishing likely continues on the AR at night (*Pers. Comm*. chair of local fishers committee). These observations support model predictions that low levels of illegal fishing might not disturb the positive impact of the AR. Alternatively surveillance effort could be supported by the community if benefits were managed according to ancestral traditions. Indeed, "no take area" regime on the AR would be in line with some past West African tribal laws, applied before the colonization era, which set marine area where fishing activities were restricted for occasional community celebrations. Collective processes where fishers and other stakeholders can design temporary no-take zones around the AR could increase fishers trust and compliance to the rules, fostering a positive socio-ecological feedback loop^30^.

Hybridization of local and scientific knowledge, through the integration of natural sciences and social sciences, is key point for governance setting^31–33^. Indeed, the communication of the resulting hybrid knowledge in specific events gathering local stakeholders helps strengthen fisheries co-management for the establishment of surveillance and regulatory frameworks. This phenomenon was experienced during the public restitution of the present study with the community, fishers, children’s from local schools and governmental stakeholders. Science popularization of the study results was in French and local language (Wolof) retransmitted on national news (available at https://www.youtube.com/watch?v=yQqFU2P4XZU). Posters were exposed during the event, including pictures of local fishers interviewed and statements reflecting their own perception of how the artificial reef interacts with ecological processes and fisheries dynamics. Straightaway, stakeholders and local promoters of AR publicly expressed their concern and willingness to prioritize the setting up an efficient AR surveillance independent from external resources prior to increase AR deployments. Knowledge hybridization could produce more specific models that could be used for warning and advice, for example by considering potential impacts of ARs on species compositions^3,34,35^, environmental parameters^36^, and cascade effects on the trophic food web^37^. However this approach would need to be adapted to local social-ecological governance, which might require dedicated political-anthropological studies (see concept of adaptive co-management^32^).

In summary, best practices should involve all stakeholders, consider local specificities, such as site configuration, governance, ecosystem, availability of ad hoc human and financial resources for AR surveillance, and define AR volume and design accordingly to these parameters. Thus, if plans exist to deploy ARs at large scales we recommend that legislation is strengthened, with detailed Environmental and social Impact Assessments^38^ to implement ARs, including considerations of long-term governance.

## Methods

### Mathematical approach

The mathematical model considered a coastal area in which fisheries are managed by the deployment of an AR in a no-take area surrounded by a fishing zone. It is a demonstration tool based on four generally accepted assumptions:

1. Fish population growth can be described using a logistic equation;
2. Fisheries landings can be described as a product between fishing effort, fish density, and a catchability coefficient;
3. Fishing effort increase or decrease according to the benefits of the fisheries;
4. Artificial reefs have both fish attraction and production effects.

Production and attraction effects of ARs were taken into account in an earlier version of the model in which illegal fishing was not incorporated^14^. In the current study, the model assumed there was some “illegal” fishing in the no-take area (Fig. 1). The model focused on assessing how illegal fishing affected local fisheries yields in and around ARs. Thus, the domain considered was the AR no-take area, plus the surrounding fishing area, in which AR spillover was likely to be perceived by fishers 15. The analysis of the model was made analytically, i.e. without making assumptions on the parameters values (See Supporting Information Sect. 1). The use of the variable aggregation method allowed us to express the possible equilibriums between fish biomass and fishing effort as a function of the parameters. Then, we chose a set of parameters that roughly fit the case study fisheries, with the exception of parameters for attraction, production and illegal fishing (i.e. rate of fishing effort applied on the AR). These final parameters were varied to estimate the sensitivity of model solutions to these parameters. Thus, the modeling approach presented here does not pretend to produce accurate numerical predictions; instead it is used to shows the existence of different possible fisheries regimes according to these parameters, one of them being directly related with management (the rate of fishing on the AR).

The fish community was represented in the model as a pool of species characterized by an average growth rate, *r*, and movement behavior, which consists of an ideal free distribution (IFD). “*a*” was the parameter determining fish mobility; the higher the value of this parameter, the faster fish biomass reached the IFD. It was assumed that the fish population was isolated from other populations (*i.e*., movements in and out of the domain were not considered). The fishery associated with this fish community was defined by the cost per unit effort, *c*, the market price, *p*, and the fish catchability coefficient, *q*; however, the latter could also be interpreted as a property of the fish. The carrying capacity of the studied area, *K,* is considered uniform prior the immersion of ARs. Based on the published literature, we formulated the hypothesis that ARs can: (1) add an additional carrying capacity per unit of AR volume, *δk*, to the area where they are deployed, accounting for the production effect; and (2) modify fish IFD between the fishing area and no-take area, through the attraction effect. Given “*V*”, the volume of ARs, and “*α*”, the proportion of the fishing area that is no-take area, the total carrying capacity of the no-take area was given as *αK* + *Vδk*. Similarly, the carrying capacity of the open access area was given as *(1-α)K*. The attraction effect was simulated by an “attraction function” (see Supporting Information 1a for details). The attraction function ($\beta$) described the part of fish movement that did not correspond to the IFD, representing the perturbation induced on fish displacement due to the attractive effect of ARs.

Changes to carrying capacity due to ARs were integrated in the population model in the logistic growth function of the population in the no-take area, while the attraction function was included in the migration term describing fish movement between no-take areas and fishing areas. *n*_*1*_ and *n*_*2*_ represented fish biomass in the no-take area and fishing area, respectively. Similarly, *E*_*1*_ and *E*_*2*_ were the corresponding fishing effort in these two areas. The evolution of fish biomass and fishing effort was described by the following ordinary differential equations (Eq. 1):

1 $$\left\{\begin{array}{l}\frac{d{n}_{1}}{d\tau }=\left(\frac{a}{\left(1-\alpha \right)K }+\beta \left({V}_{1}\right)\right){n}_{2}-\left(\frac{a}{\alpha K+{V}_{1}\delta K}\right){n}_{1}+\varepsilon \left(r{n}_{1}\left(1-\frac{{n}_{1}}{\alpha K+{V}_{1}\delta K}\right)-{q}_{1}{n}_{1}{E}_{1}\right)\\ \frac{d{n}_{2}}{d\tau }=\left(\frac{a}{\alpha K+{V}_{1}\delta K}\right){n}_{1}-\left(\frac{a}{\left(1-\alpha \right)K}+\beta \left({V}_{1}\right)\right){n}_{2}+\varepsilon \left(r{n}_{2}\left(1-\frac{{n}_{2}}{\left(1-\alpha \right)K}\right)-{q}_{2}{n}_{2}{E}_{2}\right)\\ \frac{d{E}_{1}}{d\tau }={m}_{1}{E}_{2}-{m}_{2}{E}_{1}+\varepsilon \left(p{q}_{1}{{E}_{1}n}_{1}-c{E}_{1}\right)\\ \frac{d{E}_{2}}{d\tau }={m}_{1}{E}_{1}-{m}_{2}{E}_{2}+\varepsilon \left(p{q}_{2}{{E}_{2}n}_{2}-c{E}_{2}\right)\end{array}\right.$$

Note, here, $\tau$ stands for fast time scales (*e.g*., hours), and ε <  < 1 stands for a small dimensionless parameter with the purpose of discriminating “slow” and “fast” time scale processes. Growth and fishing mortality were assumed to occur at a slow time scale compared to fish and boat movement.

Assuming the fast movement of fish and boats between the two zones, we derived a reduced model^39–42^ for total fish biomass n(t) and fishing effort E(t) (Eq. 2):

2 $$\left\{\begin{array}{l}\frac{dn}{dt}=rn\left(1-\frac{{{\nu }_{1}^{*}}^{2}n}{\alpha K+{V}_{1}\delta K}-\frac{{(1-{\nu }_{1}^{*})}^{2}n}{\left(1-\alpha \right)K}\right)-q{\gamma \nu }_{1}^{*}nE-q{\left(1-\gamma \right)(1-\nu }_{1}^{*})nE\\ \frac{dE}{dt}=\left(pq{\gamma \nu }_{1}^{*}n+pq{\left(1-\gamma \right)(1-\nu }_{1}^{*})n-c\right)E\end{array}\right.$$

here *t* stands for slow time scales (*e.g.,* months). See Supporting Information Sect. 1 for the presentation of the complete model and reduction method.

Using this model, we looked for the effects of illegal fishing (given by parameter γ, which was the fraction of total fishing effort deployed in the no-take area) for different volumes of ARs under different attraction and production conditions. The parameter values used here (Table 1) were set so that the area considered had a carrying capacity of 100 tons of fish. 20% of this area was considered as the AR no-take area, and the rest was fishing area. Thus, as the no-take area around the Yenne AR had a radius of 500 m, the fishing area considered was 618 m around the no take area. This distance was large enough to include the average distance of the no take area in which spillover was perceived by fishers [~ 200 m around the no-take area^15^], at least for territorial AR-associated species, such as *Epinephelus costae*, which is one of the species inhabiting the AR with the highest commercial value^16^*.*

**Table 1:** Values of the parameters in the mathematical model describing changes to fishing effort and fish population growth depending on the volume of the artificial reef and distribution of fishing effort.

Parameter	Value used	Description
*c*	1	Fishing cost per unit effort
*p*	1	Fish price
*q*	1	Fish catchability
*K*	100	Fish carrying capacity of the environment
*r*	0.5	Fish growth rate
*a*	2	Fish mobility
*γ*	0–1	Distribution of Fishing effort between fishing area and artificial reef
$\alpha$	0.2	Ratio of MPA
$\beta_{0}$	0.1; 1	AR attraction parameter 1
$\sigma$	0.1	AR attraction parameter 2
$\delta K$	0.1; 5	Fish carrying capacity per AR

*MPA* Marine Protected Area, *AR* Artificial Reef.

### Social field survey

The mathematical model was complemented by a field survey in 2014 to elucidate the perceptions of fishers on the effect of an AR and its management. The survey presented here was designed to capture the perceptions of fishers on the ecological function of the AR and its effect on local fisheries dynamics based on the key biological processes also included in the mathematical model (attraction, production, and spill over). The survey also documented the knowledge of fishers and the extent to which they adhere to actual rules in the co-management of the AR. The survey started with a meeting of the seven village chiefs and notables who helped facilitate the survey at seven locations near the AR (Yenne Municipality communities, Table 2) and were able to provide information on past actions and present statutes. Interviews were then conducted over a 10-day period from June 24, 2014 (Table 2). Only fishers that were actively fishing before the AR was immersed were interviewed. Interviewed fishers were randomly chosen directly at their landing sites. The sample was stratified by the age (Fig. S6), experience, fishing techniques used (with four fishing techniques being actively practiced in the study area; Fig. S7), status (*e.g*., captain, sailor, ship owner), and affiliation (or not) of fishers to their local fishery committee (*i.e*., eco-management organizations for artisanal fishers). Ten fishers per village were interviewed separately, totaling seventy interviews across the seven villages surveyed. The questionnaire and field report, as well as a data table on which all responses were compiled, are provided in the supplementary data.

**Table 2:** Interviewed fishers for the sociological study conducted on June 24, 2014 in the seven village of the Yenne Municipality communities located ~ 30 km south of Dakar (Senegal).

Village	Distance to artificial reef (km)	Main Fishing gear	Mean age (year; min. – max.)	Affiliated to a fisher organization (%)
Yenne Tod	3.8	Gill net, Line, Longline	48 (30–77)	40
Yenne Guedj	3.6	Gill net, Longline	47 (31–57)	70
Yenne Kao	3.5	Gill net, Longline	53 (43–63)	60
Yenne Nditakh	3.7	Gill net, Purse Seine	53 (28–72)	60
Yenne Nianghal	4.2	Gill net, Line, Longline	54 (37–72)	30
Kelle	4.6	Line, Longline, Gill net	39 (26–50)	10
Toubab Dialaw	5.3	Line, Longline, Gill net	38 (25–69)	0

A sample of ten (10) fishers were interviewed in seven village where fishers operate around the artificial reef deployed at 14°37.032'N, 17°12.007'W. The distance from the artificial reef of each village is displayed, the main fishing gear used by the fishers and their average age, and the percentage of interviewed fisher affiliated to fisheries management body.

The questionnaire consisted of 36 questions. The first 10 questions gathered information about the interviewed fishers, including their social position among fishers, experience, fishing gear used, and favored target fish species. Then, five multiple choice general questions were given to assess respondent opinions on why fish depletion occurred in the area, the acceptance of management measures, changes to catches perceived after AR implementation, and property perception of the fish present in the AR. Four further questions were asked about the limits and characteristics of the coastal area where the respondents fished, and how the fishing area is shared with others fishers. Finally, 17 semi-open and open questions focused on the ecological processes of fish attraction, production, and spill over (*i.e.,* the basic fisheries dynamics implemented in the mathematical model). For details on the social survey, see the Supporting Information Sect. 2.

### Ethic statements

The studies involving human participants were reviewed and approved by both the Senegalese center of oceanographic research (ISRA/CRODT) and the Environmental and sustainable development Senegalese Ministry (MEDD). The experimental protocol (survey) was reviewed and approved by both national institutions. All participants to the survey did it voluntary. Written informed consent for participation was not required for this study in accordance with the national legislation and the institutional requirements.

## Supplementary Information

Supplementary Information.
