# Publisher Correction: Successful artificial reefs depend on getting the context right due to complex socio-bio-economic interactions

**DOI:** 10.1038/s41598-021-97724-3

**Published:** 2021-09-06

**Authors:** Timothée Brochier, Patrice Brehmer, Adama Mbaye, Mamadou Diop, Naohiko Watanuki, Hiroaki Terashima, David Kaplan, Pierre Auger

**Affiliations:** 1 UMMISCO, Sorbonne Université, SU, Institut de Recherche pour le Développement, IRD, 93143 Bondy, France; 2 UMMISCO, Université Cheikh Anta Diop, UCAD, Ecole Supérieure Polytechnique, ESP, IRD, BP 15915, Dakar, Senegal; 3 Ifremer, CNRS, IRD, Univ Brest, Plouzané, Lemar, France; 4 Centre de Recherche Océanographique de Dakar‑Thiaroye, CRODT, Institut Sénégal de Recherches Agricoles, ISRA, Dakar, Senegal; 5 Directorate of Community Marine Protected Areas, DAMCP, Ministry of the Environment and Sustainable Development, MEED, Dakar, Senegal; 6 OAFIC, 2F Kanda 4th Amelex Bldg., 2‑13 Kanda Tsukasamachi, Chiyodaku, Tokyo, 101‑0048 Japan; 7 IC Net Limited, Land Axis Tower 27th Floor, 11‑2 Shintoshin, Chuo‑ku, Saitama, 330‑6027 Japan; 8 MARBEC, Univ. Montpellier, CNRS, Ifremer, IRD, Sète, France; 9 IRD, MARBEC, av. Jean Monnet, CS30171, 34203 Sète cedex, France

Correction to: *Scientific Reports* https://doi.org/10.1038/s41598-021-95454-0, published online 17 August 2021

The original version of this Article contained an error in Affiliation 8, which was incorrectly given as ‘CNRS, Ifremer, IRD, MARBEC, Univ. Montpellier, Montpellier, France’.

The correct affiliation is listed below.

MARBEC, Univ. Montpellier, CNRS, Ifremer, IRD, Sète, France.

The original Article has been corrected.

