# Supplementary material for: Successful artificial reefs depend on getting the context right due to complex socio-bio-economic interactions
Source: Sci Rep. 2021 Aug 17;11:16698. doi: 10.1038/s41598-021-95454-0 (PMC8371003; doi:10.1038/s41598-021-95454-0)
Supplement: Supplementary file 1 — Supplementary Information. [file 41598_2021_95454_MOESM1_ESM.pdf]

Supporting Information for

Artificial reefs: a double-edged fishery management tool in poor governance areas

Timothée Brochier, Patrice Brehmer Pierre Auger, and Adama Mbaye

Correspondence to: timothee.brochier@ird.fr

Supporting Methods

1- Mathematical model: the theoretical effect of fishing on the artificial reef **2**
a. Model hypotheses 2
b. The complete model description 3
c. Fast equilibrium 4
d. The aggregated model 5
e. Analysis of the aggregated model 6
2 - Local knowledge: considering the actors point of view **1**
a. Context and study area **1**
b. Survey methods **2**
Fig. S1. . .... **3**
Fig. S2.. .... **4**
Fig. S3.. .... **5**
Fig. S4.. .... **9**
Fig. S5. .... **10**
Data S1. (separate file) **11**
Data S2. (separate file) **11**

1- *Mathematical model: the theoretical effect of fishing on the artificial reef*

a. Model hypotheses

Let us consider a coastal area where fisheries are managed by the deployment of a volume of artificial habitats or artificial reefs (ARs), both referring to the same thing hereafter. In principle, the area where ARs are deployed is a no-take area, however due to management deficiencies and illegal fishing, it is usual that a proportion of the fishing effort occurs directly on the area where artificial reefs are located. Let 'K' be the total fish carrying capacity of the coastal area, which is uniform. The area is divided into two parts for fisheries management, the exact area were ARs are deployed, which has a carrying capacity of αK (prior to the ARs deployment), which is supposed to be a no-take zone, *i.e.*, a marine protected area (MPA), and the surrounding area, *i.e.*, the normal fishing grounds with a carrying capacity (1-α) K where α is the proportion of the total area where ARs are deployed.

The model considers the fish community as a whole; which can be represented as a pool of species sharing the same logistic growth with a growth rate, r, and the same movement behavior. The fish population is assumed to be isolated from other populations, *i.e.*, there are no migrations out of the domain considered. The fishery associated with this fish community is defined by the cost per unit effort, c, the market price, p, and the fish catchability coefficient, q, although the latter can also be seen as a property of the fish.

On the basis of the literature, we formulate the hypothesis that ARs can (1) add an additional carrying capacity per unit of volume, δk, to the area where they are deployed, accounting for the production effect, and (2) modify the fish ideal free distribution (IFD) between the fishing area and the MPA, as a result of the attraction effect. Let "V" being the volume of ARs, thus the total carrying capacity of the MPA is given by αK + Vδk. Similarly, the carrying capacity of the fishing area is given by (1-α) K. The attraction effect was simulated by an "attraction function" of the volume of ARs, *β(V),* which is an extra migration rate from the fishing area toward the ARs (eq. 1).

$$\beta(V) = \left(\frac{\beta_0 V}{1+\sigma V}\right) \quad \text{(eq. 1)}$$

Where β_0 and σ are parameters describing the shape of the curve. This function is strictly positive and monotone increasing with respect to V and displaying a plateau equal to $\frac{\beta_0}{\sigma}$. σ describes the steepness of the attraction function $\beta(V)$ with respect to V. In the absence of attracting effect (β_0= 0), our model considers an ideal free distribution (IFD) of the fish, *i.e.* an optimal distribution of the fish according to the distribution of carrying capacity. The attraction function (eq. 1) describes the part of fish movement that does not correspond to the IFD, but represents the perturbation induced by fish displacement due to the purely attractive effect of the ARs.

b. *The complete model description*

Let n_1 and n_2 respectively being the fish biomass in the MPA (where the ARs are deployed) and in the fishing area, as well as E_1 and E_2 the corresponding fishing efforts. The evolution of the fish biomass and fishing effort is described by the following ordinary differential equations (eq. 2):

$$\begin{cases} \frac{dn_1}{d\tau} = \left(\frac{a}{(1-\alpha)K} + \beta(V_1)\right) n_2 - \left(\frac{a}{\alpha K + V_1 \delta K}\right) n_1 + \varepsilon\left(r n_1 \left(1 - \frac{n_1}{\alpha K + V_1 \delta K}\right) - q_1 n_1 E_1\right) \\ \frac{dn_2}{d\tau} = \left(\frac{a}{\alpha K + V_1 \delta K}\right) n_1 - \left(\frac{a}{(1-\alpha)K} + \beta(V_1)\right) n_2 + \varepsilon\left(r n_2 \left(1 - \frac{n_2}{(1-\alpha)K}\right) - q_2 n_2 E_2\right) \\ \frac{dE_1}{d\tau} = m_1 E_2 - m_2 E_1 + \varepsilon(p q_1 E_1 n_1 - c E_1) \\ \frac{dE_2}{d\tau} = m_1 E_1 - m_2 E_2 + \varepsilon(p q_2 E_2 n_2 - c E_2) \end{cases} \quad \text{(eq. 2)}$$

With $\varepsilon \ll 1$ is a small dimensionless parameter. τ is the fast time.

Equations 2 are called the complete model as they take into account processes going on at different time scales. They are composed with two parts, a fast one and a

slow one. The fast part relates to fish and fishermen migrations between the MPA and the fishing area while fish growth, landings and fishing effort (investment in fishing means) vary at the slow time $t=\varepsilon\tau$. Slow terms of the complete models are those multiplied by ε and correspond to fish growth, fishing mortality and fishing effort. For slow-fast models, we refer to the early work of Tikhonov (1952). Perfect and approximate methods of aggregation of variables were defined in (Iwasa, Andreasen, & Levin, 1987; Iwasa, Levin, & Andreasen, 1989). Auger et al. (2008) have developed methods of aggregation of variables in dynamical systems with different time scales based on a Fenichel version of the center manifold theorem.

In our case, we use "aggregation methods". A first step of the method is to look for the existence of an asymptotically stable equilibrium of the fast part of the complete model. In a second step, we make an approximation by substituting the fast variables in terms of the fast equilibrium into the complete model. This allows us to get a reduced model, also called the aggregated model, governing slow variables at a slow time scale.

c. *Fast equilibrium*

By setting $\varepsilon=0$, small perturbation terms vanish in the complete model and we get the fast system which in our case corresponds only to fish and boat migrations between the MPA and the fishing area. Because migration is conservative, $n = n_1 + n_2$ and $E = E_1 + E_2$ are constant at the fast time. In our case, the fast model reads as follows (eq. 3):

$$\begin{cases} \frac{dn_1}{d\tau} = \left(\frac{a}{(1-\alpha)K} + \beta(V_1)\right)n_2 - \left(\frac{a}{\alpha K+V_1\delta K}\right)n_1 \\ \frac{dn_2}{d\tau} = \left(\frac{a}{\alpha K+V_1\delta K}\right)n_1 - \left(\frac{a}{(1-\alpha)K} + \beta(V_1)\right)n_2 \\ \frac{dE_1}{d\tau} = m_1E_2 - m_2E_1 \\ \frac{dE_2}{d\tau} = m_1E_1 - m_2E_2 \end{cases} \qquad \text{(eq. 3)}$$

In our case, the total fish biomass $n=n_{1+}\ n_2$ and fishing effort $E=\ E_{1+}\ E_2$ are constant at the fast time. To get the fast equilibrium, we express the fast variables n_2=n-

n_1 in terms of the “constant” n. A straightforward calculation leads to the fast equilibrium for fish as follows (eq. 4).

$$\begin{cases} n_1^* = \nu_1^* n \\ n_2^* = \nu_2^* n \end{cases} \qquad \text{(eq. 4)}$$

Where v_1* and v_2*=1- v_1* represent the asymptotic spatial distribution of individuals respectively in zone 1 and 2 due to fast fish migration. They are given by the following expressions (eq. 5):

$$\begin{cases} \nu_1^* = \dfrac{\frac{a}{(1-\alpha)K}+\beta(V_1)}{\frac{a}{(1-\alpha)K}+\beta(V_1)+\frac{a}{\alpha K+V_1\delta K}} \\ \nu_2^* = \dfrac{\frac{a}{\alpha K+V_1\delta K}}{\frac{a}{(1-\alpha)K}+\beta(V_1)+\frac{a}{\alpha K+V_1\delta K}} \end{cases} \qquad \text{(eq. 5)}$$

Similarly, for the fishing effort, we get the next expressions at the fast equilibrium:

$$\begin{cases} E_1^* = \gamma E \\ E_2^* = (1-\gamma)E \end{cases} \qquad \text{(eq. 6)}$$

With

$$\gamma = \frac{m_1}{m_1+m_2} \qquad \text{(eq. 7)}$$

Where γ corresponds to the fraction of the total fishing effort that is deployed in the MPA at the fast equilibrium. In other words it represents the share between legal and illegal fishing effort.

d. The aggregated model

The next step is to make an approximation by substituting the fast variables in terms of the fast equilibrium (n_1^*, n_2^*, E_1^*, E_2^*) into the equations of the complete model. Adding

fish and boat equations, and using slow time t, we obtain a reduced model, called the "aggregated model", which reads as follows (eq. 8):

$$\begin{cases} \frac{dn}{dt} = rn\left(1 - \frac{{v_1^*}^2 n}{\alpha K + V_1 \delta K} - \frac{(1-v_1^*)^2 n}{(1-\alpha)K}\right) - q\gamma v_1^* nE - q(1-\gamma)(1-v_1^*)nE \\ \qquad \frac{dE}{dt} = (pq\gamma v_1^* n + pq(1-\gamma)(1-v_1^*)n - c)E \end{cases} \quad \text{(eq. 8)}$$

This "quick derivation method" is valid when the aggregated model is structurally stable, that is the case of the aggregated model used in this study (eq. 8). Also, the parameter ε must be small enough. When all parameters of the complete model are of the same order, for instance of the order 1, numerical simulations performed for previous examples have shown that parameter ε should be at least smaller than 10^{-1}, see for examples of numerical simulations in ***(Error! Reference source not found.)***. Under these conditions, trajectories starting at the same initial condition for the complete and aggregated models remain close of each other.

e. Analysis of the aggregated model

The aggregated model can be rewritten as follows (eq. 9):

$$\begin{cases} \frac{dn}{dt} = rn\left(1 - \frac{n}{K_{ag}}\right) - q\gamma v_1^* nE - q(1-\gamma)(1-v_1^*)nE \\ \quad \frac{dE}{dt} = (pq\gamma v_1^* n + pq(1-\gamma)(1-v_1^*)n - c)E \end{cases} \quad \text{(eq. 9)}$$

where K_{ag} is the total carrying capacity of the fish population on the two zones, MPA and fishing, which is defined as follows :

$$\frac{{v_1^*}^2}{\alpha K + V\delta K} + \frac{(1-v_1^*)^2}{(1-\alpha)K} = \frac{1}{K_{ag}} \quad \text{(eq. 10)}$$

The aggregated model is of the same form as the classical Lotka-Volterra predator-prey model with prey logistic growth. Consequently, we know that there exist only three equilibria: (0, 0), $(K_{ag}, 0)$ and (n^*, E^*) where :

$$n^* = \frac{c}{pq(\gamma v_1^* + (1-\gamma)(1-v_1^*))} \qquad \text{(eq. 11)}$$

$$E^* = \frac{r}{q(\gamma v_1^* + (1-\gamma)(1-v_1^*))}\left[1 - \frac{v_1^{*2}}{\alpha K + V\delta K} n^* - \frac{(1-v_1^*)^2}{(1-\alpha)K} n^*\right] \qquad \text{(eq. 12)}$$

We know that when equilibrium (n^*, E^*) is positive, it is globally asymptotically stable in the positive quadrant. Under these conditions, we are going to study the sensitivity of the catch in the fishing area and in the MPA (illegal fishing) at equilibrium with respect to different values of V (AR volume) and γ (the proportion of illegal fishing).

In figures S1, S2 and S3, we respectively plot the equilibrium catch in the fishing area (Y_2^* eq. 13), in the MPA (Y_1^* eq. 14), and the total catch ($Y_1^* + Y_2^*$). We compare four cases according to model assumptions on ARs productivity (δK = 0.1 ; 5) and attraction (β_0= 0.1; 1) (Fig. S1, S2; S3).

$$Y_2^* = q(1-\gamma)(1-v_1^*)n^*E^* \qquad \text{(eq. 13)}$$

$$Y_1^* = q\gamma v_1^* n^* E^* \qquad \text{(eq. 14)}$$

2 - Local knowledge: considering the actors point of view

a. Context and study area

Yenne and Bargny are two coastal fishing territories in Senegal in which artificial reef was deployed in 2004 in the frame of the Japanese collaboration (JICA). In January 2012, a sociologic survey carry out by the Senegalese fisheries and oceanographic center "CRODT" (Mbaye, 2012) in Yenne and Bargny revealed that 95% and 85%, respectively, of the artificial reef area was "lethargic". The fact was that since the end of the JICA project that initiated the reef, monitoring, control and surveillance was no longer performed. The survey suggested that at the community level, the establishment of protected fishing areas and artificial reef areas requires strict monitoring to ensure compliance with management rules. As a result, the lack of monitoring capacity was identified as the main factor that can undermine incentives in MPAs and artificial reef areas.

The lack of cooperation from the fisheries administration was cited as the first factor in non-compliance with management measures. This factor was particularly mentioned in Yenne with 75% of fishermen saying that since the end of the project with JICA, they have not received any support from the fisheries administration to continue monitoring as well as control and surveillance of the artificial reef area.

In 2014, we performed a second study, directly designed to collect the fishermen point of view of the ecological – management interactions effects of the artificial reef, in order to complete the mathematical approach performed (Mbaye, 2012). Thus, fisheries sociologist in close collaboration with mathematicians and fisheries scientist established an *ad hoc* questionnaire.

b. Survey methods

The questionnaire was built with the idea of collecting fishermen perception of the ecological function of the artificial reefs and their effect on the local fisheries dynamics. A first part of the questionnaire was information about the interviewed fishermen, in particular his social position among fishermen, experience, fishing gear and favorite targets. Only fishermen that were actively fishing before the reef was immersed were interviewed. The second part of the questionnaire was designed to evaluate the knowledge and practice of actual rules in the current co-management of the artificial reef. Finally, semi-open and open questions were designed to capture the fishermen perception and understanding of the ecological processes of attraction, production and spill over and basic fisheries dynamics as implemented in the mathematical model.

The study was conducted on the artificial reef of Yenne territory containing the villages of Yenne Todd, Yenne Guedj, Yenne Kao, Yenne Nditakh, Nianghal, Yenne Kell and Touba Dialaw. For data collection, interviews were conducted with the identified actors following classic sociology survey methods (Dufour, Fortin, & Hamel, 1991; Olivier de Sardan, 1995; Paugam, 2012). Interviewed fishermen were randomly chosen directly on their small scale landing sites. The resulting sample displayed a good diversity in interviewed fishermen age (Fig. S4), experience, fishing technique used (limited at 4 in the study area; Fig. S5), status (e.g., captain, sailor, ship owner), and affiliation or not to local fisheries co-management organizations. The interviews consisted of individual interviews by questionnaire. Ten fishermen per village were interviewed for a total of seventy. The questionnaire and field report of the survey in French is provided as online supplementary data, as well as an Excel sheet that compile all collected responses.

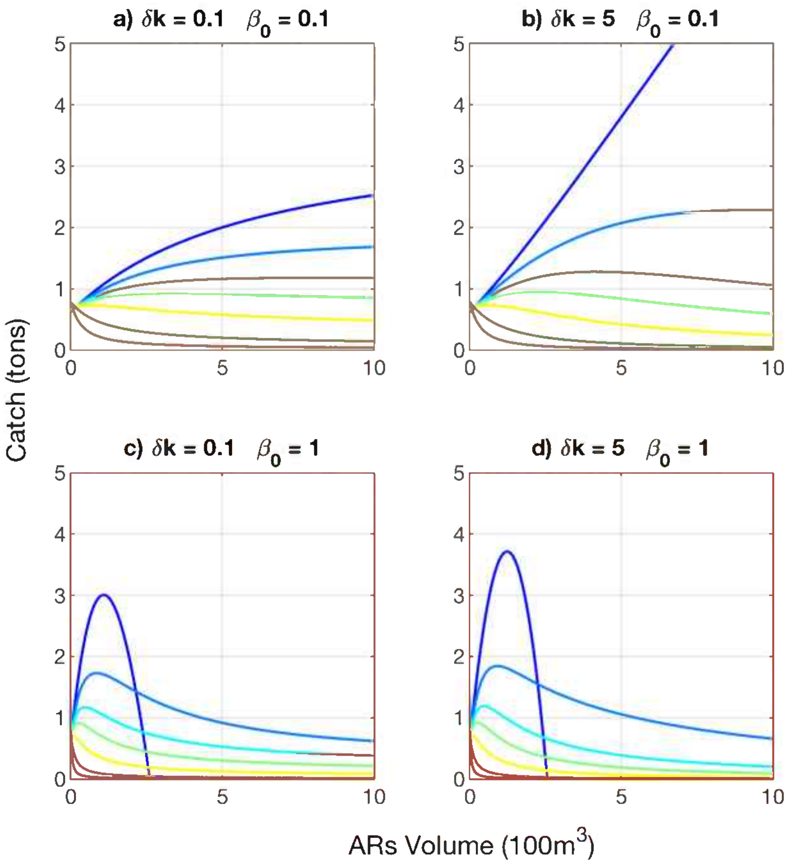

Fig. S1. Effect of increasing the volume of artificial reefs (ARs) on the catch in the fishing area in the case of ARs with: a) low production and attraction effect; b) strong production but low attraction effect; c) low production but strong attraction effect; d) strong production and attraction effects. The colours correspond to different levels of illegal fishing effort over the ARs. From dark blue to orange respectively 0%, 5%, 10%, 25%, 50%, 75% and 100% of illegal fishing effort. MPA: Marine Protected Area. δK: additional carrying capacity per unit of volume. β_0: Strength of the AR attraction effect from the MPA toward the ARs.

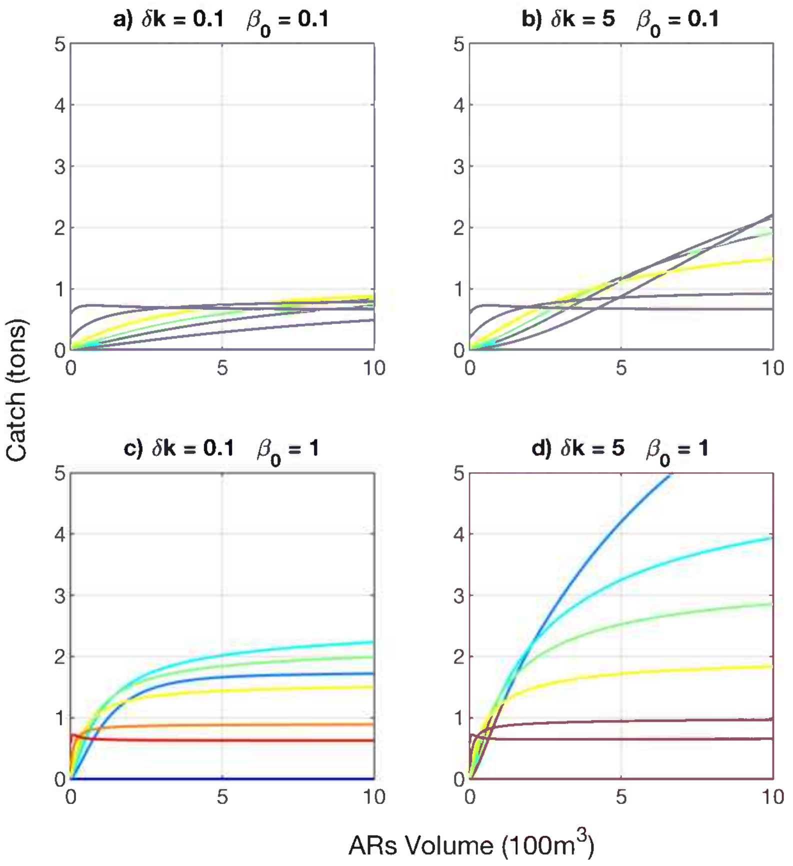

Fig. S2. Effect of increasing the volume of artificial reefs (ARs) on the illegal catch in Marine Protected Area, noted MPA, (where AR are deployed) in the case of ARs with: a) low production and attraction effect; b) strong production but low attraction effect; c) low production but strong attraction effect; d) strong production and attraction effects. The colours correspond to different levels of illegal fishing effort over the ARs. From dark blue to orange respectively 0%, 5%, 10%, 25%, 50%, 75% and 100% of illegal fishing effort. δK: additional carrying capacity per unit of volume. β_0: Strength of the AR attraction effect from the MPA toward the ARs.

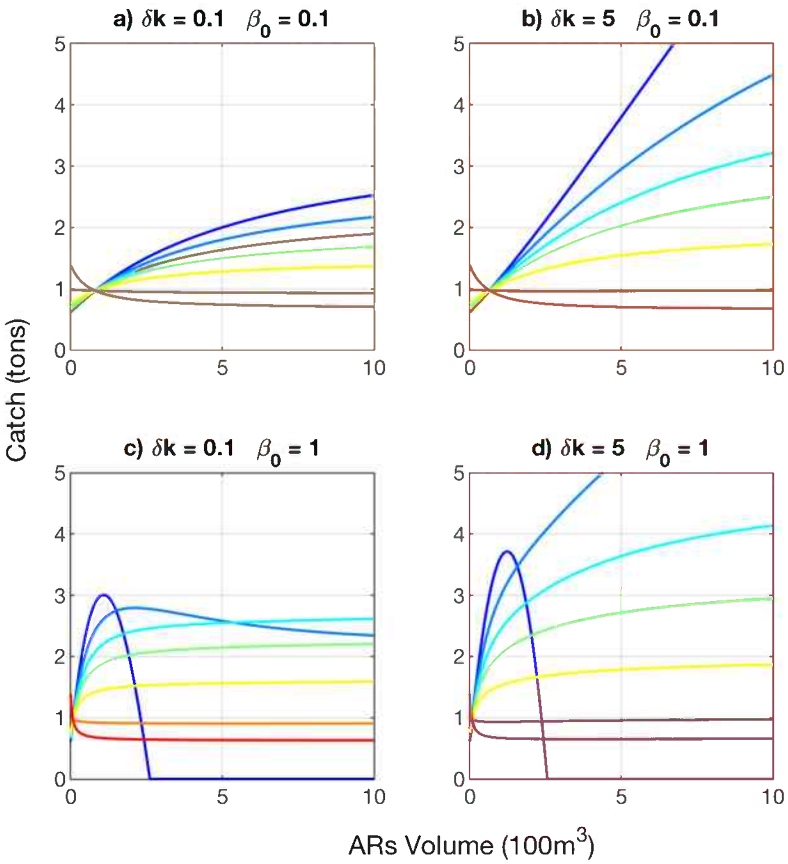

Fig. S3. Effect of increasing the volume of artificial reefs (ARs) on the total catch realized in the area (illegal catch in Marine Protected Area (MPA) and fishing area) in the case of ARs with: a) low production and attraction effect; b) strong production but low attraction effect; c) low production but strong attraction effect; d) strong production and attraction effects. The colours correspond to different levels of illegal fishing effort over the ARs. From dark blue to orange respectively 0%, 5%, 10%, 25%, 50%, 75% and 100% of illegal fishing effort. δK: additional carrying capacity per unit of volume. β_0: Strength of the AR attraction effect from the MPA toward the ARs.

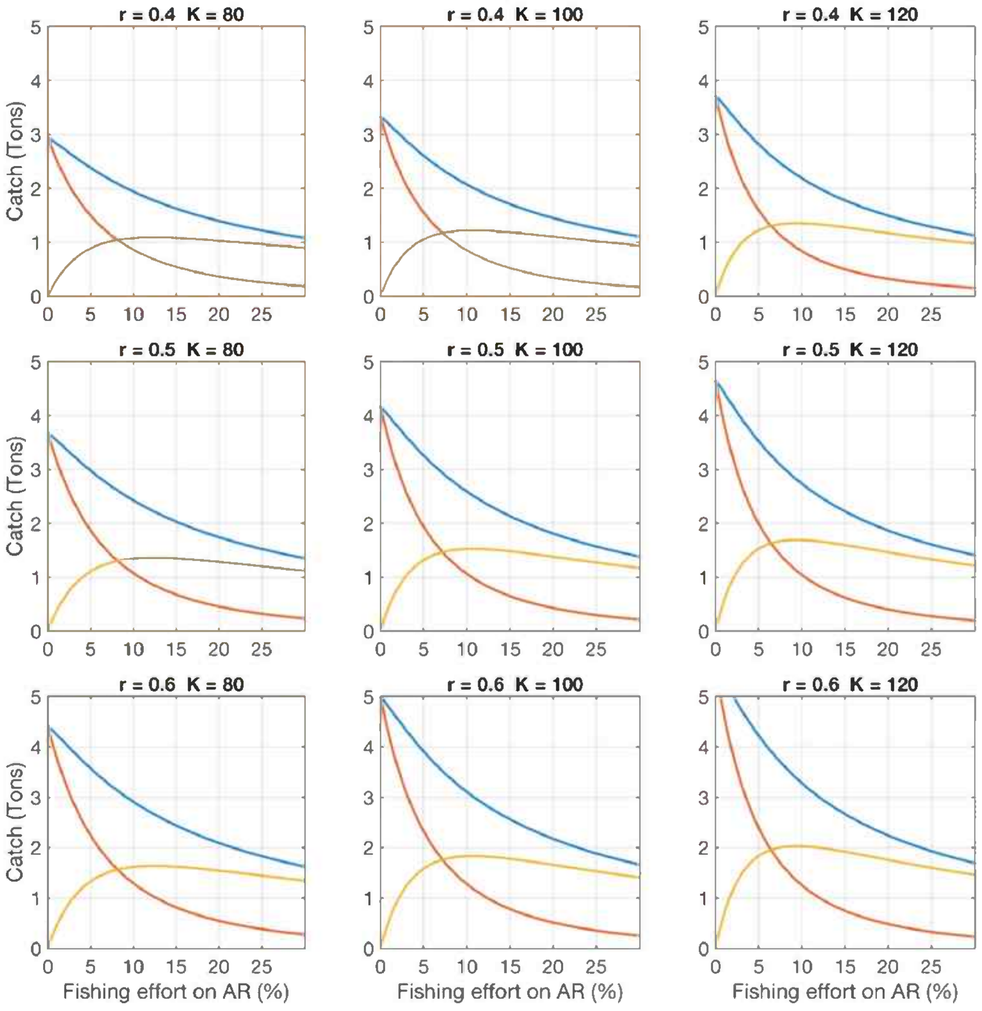

Fig. S4: Effect of variable population growth parameters (r and K) on catches predicted by the mathematical model (Y-axis) for different levels of fishing effort on AR (X-axis). Images from left to right correspond to an increasing r, and from top to bottom correspond to an increasing K. Blue, red, and yellow curves correspond to total catch, catch in the fishing area, and catch on the AR, respectively. The AR volume, production and attraction parameters were respectively set to 5, 0.5 and 200 m^3. The other parameters were set to the value given in table 1.

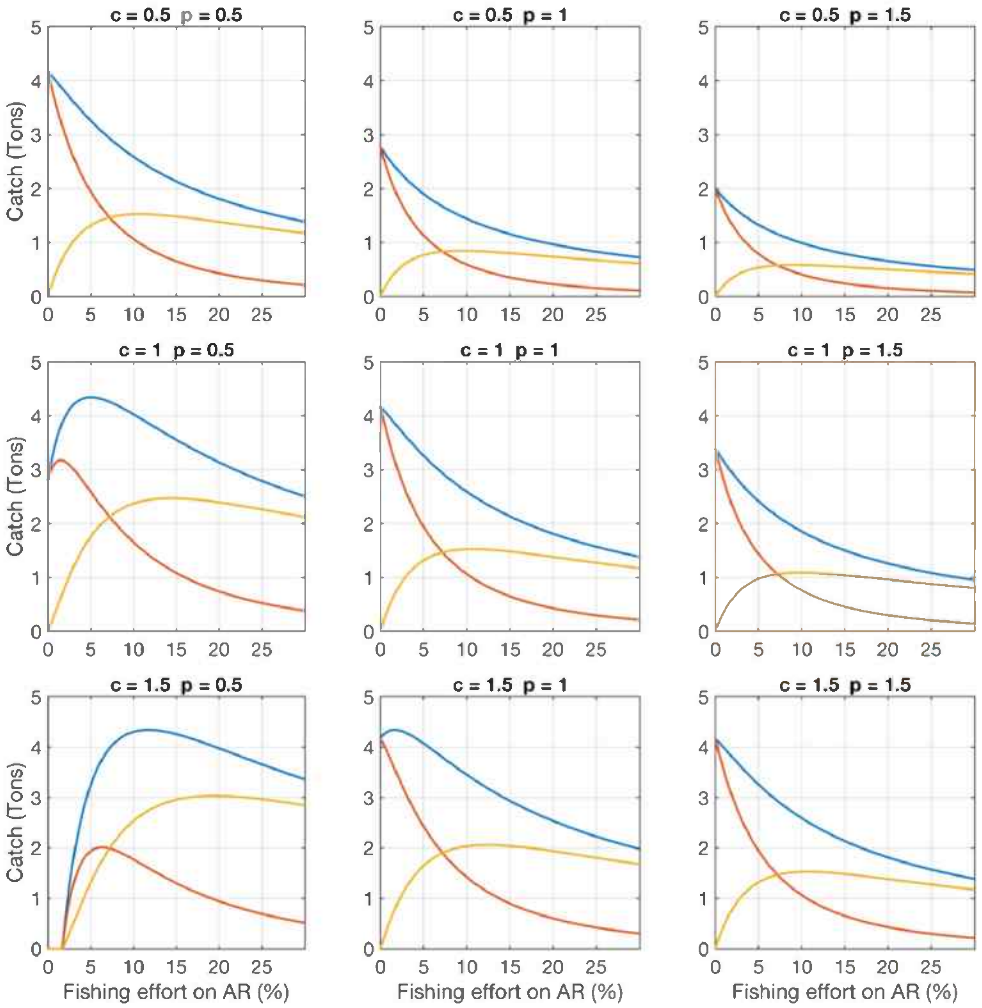

Fig. S5: Effect of variable economic parameters (c and p) on catches predicted by the mathematical model (Y-axis) for different levels of fishing effort on AR (X-axis). Images from left to right correspond to an increasing p, and from top to bottom correspond to an increasing c. Blue, red, and yellow curves correspond to total catch, catch in the fishing area, and catch on the AR, respectively. The AR volume, production and attraction parameters were respectively set to 5, 0.5 and 200 m^3. The other parameters were set to the value given in table 1.

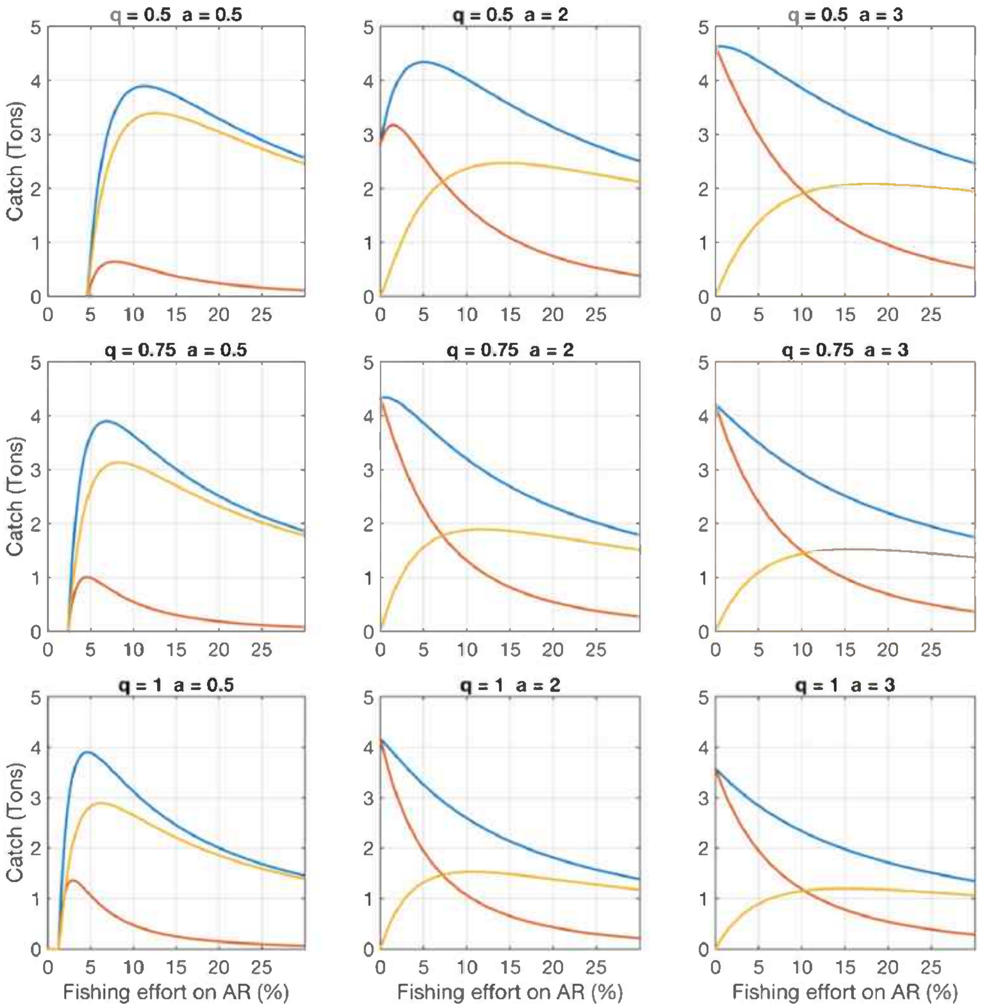

Fig. S6: Effect of variable catchability and fish mobility parameters (q and a) on catches predicted by the mathematical model (Y-axis) for different levels of fishing effort on AR (X-axis). Images from left to right correspond to an increasing a, and from top to bottom correspond to an increasing q. Blue, red, and yellow curves correspond to total catch, catch in the fishing area, and catch on the AR, respectively. The AR volume, production and attraction parameters were respectively set to 5, 0.5 and 200 m^3. The other parameters were set to the value given in table 1.

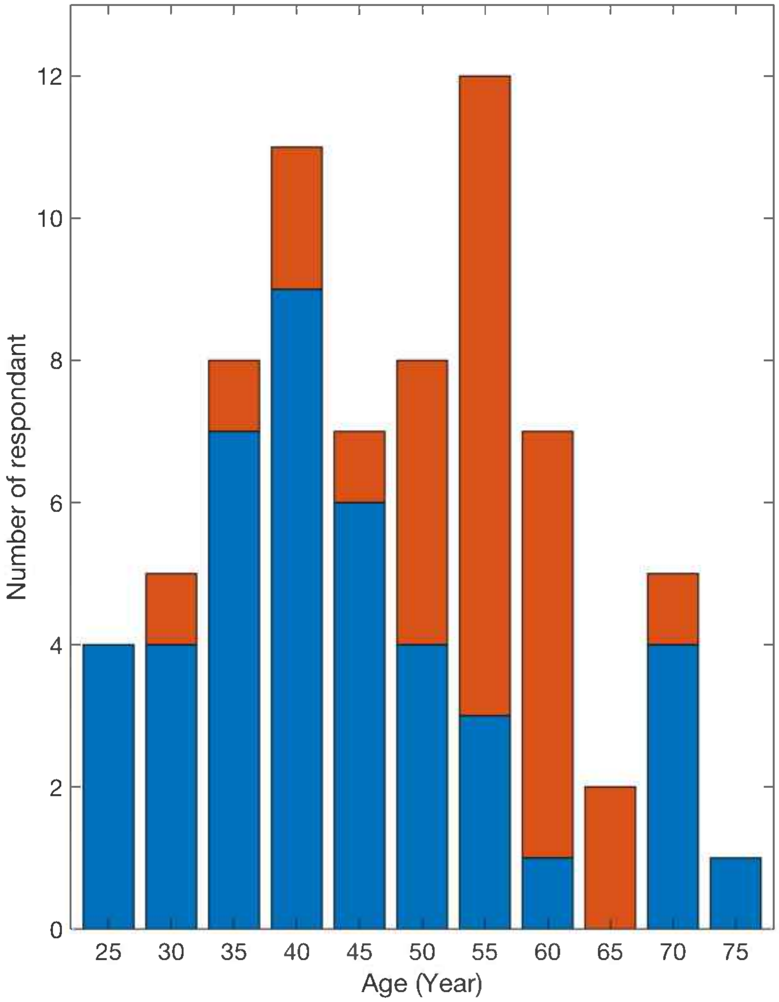

Fig. S7. Age distribution of the interviewed fishermen. The number of individuals affiliated to a local fishermen organisation (in charge of local co-management) is shown as the red part of the bars. We reported stronger participation by the older than 45 year in the fishermen organisation, also reflecting their more long-term vision towards sustainable fisheries management.

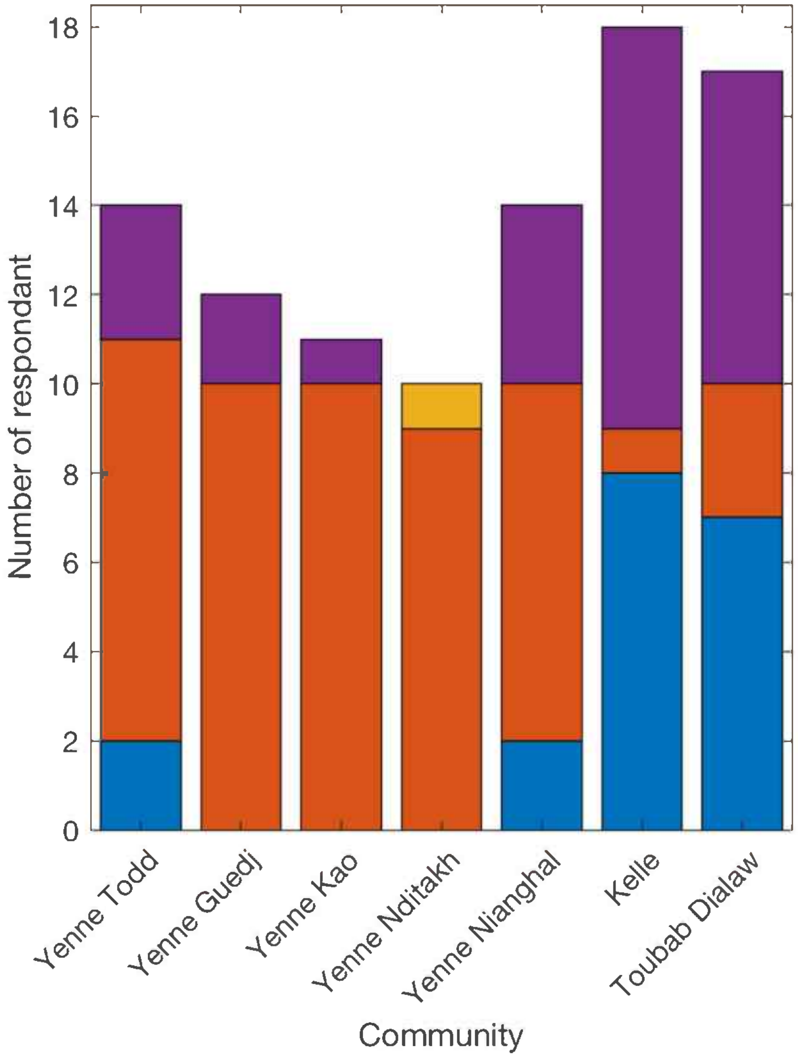

Fig. S8. Diversity of fishing practices of interviewed fishermen, per fishermen community. Community names correspond to the seven coastal localities of the Yenne commune located ~ 30km south of Dakar, Senegal. Blue: line; red: gill nets; yellow: purse senne; violet: longline.

Data S1. (separate file)

Field report of the survey on artisanal fishermen perception on artificial reef effects on fisheries and ecosystem, including the questionnaire (translated from French).

Data S2. (separate file)

Extraction of the artisanal fishermen answers (converted in modalities) during the survey on artisanal fishermen perception on artificial reef effects on fisheries and ecosystem performed on June 2014 in Yenne, Senegal (translated from French).
